# Microvascular Rarefaction and Heart Failure With Preserved Ejection Fraction

**DOI:** 10.3389/fcvm.2019.00015

**Published:** 2019-02-28

**Authors:** Heng Zeng, Jian-Xiong Chen

**Affiliations:** Department of Pharmacology and Toxicology, University of Mississippi Medical Center, Jackson, MS, United States

**Keywords:** heart failure with preserved ejection fraction (HFpEF), Sirtuin 3 (SIRT3), microvascular rarefaction, coronary flow reserve (CFR), endothelial glycolysis, heart failure with reduced ejection fraction (HFrEF)

## Abstract

Heart failure with preserved ejection fraction (HFpEF) is characterized by diastolic dysfunction and is commonly seen in the elderly and diabetic and hypertensive patients. Despite its rising prevalence, the pathophysiology of HFpEF is poorly understood and its optimal treatment remains undefined. Recent clinical studies indicate that coronary microvascular rarefaction (reduced myocardial capillary density) with reduced coronary flow reserve (CFR) is a major contributor to diastolic dysfunction in HFpEF patients. On a molecular level, endothelial cells (EC) are dependent on glycolysis for supporting their functions and vascular homeostasis. Sirtuin 3 (SIRT3) has a critical role in the regulation of endothelial glycolytic metabolism and thus affects angiogenesis. Disruption of SIRT3-mediated EC metabolism and impairment of angiogenesis may promote cardiomyocyte hypoxia and myocardial fibrosis, leading to diastolic dysfunction and HFpEF. This review summarizes current knowledge of SIRT3 in EC metabolism, coronary microvascular rarefaction and HFpEF.

## Introduction

Heart failure (HF) is a leading cause of death in the United States and worldwide. HF is a progressive disease that develops with advanced age, hypertension and diabetes. Each year over 600,000 patients are diagnosed with HF in the United States. More than half of these patients are diagnosed as heart failure with preserved ejection fraction (HFpEF) (1, 2). Diastolic function is significantly impaired in HFpEF as well in patients with heart failure with reduced ejection fraction (HFrEF) (1–5). While the standard of care for HFrEF is well-established and effective, these therapies have not shown any significant benefit for patients with preserved ejection fraction (6). Therefore, it is urgent to identify new target for the treatment of HFpEF.

**Figure 1:**
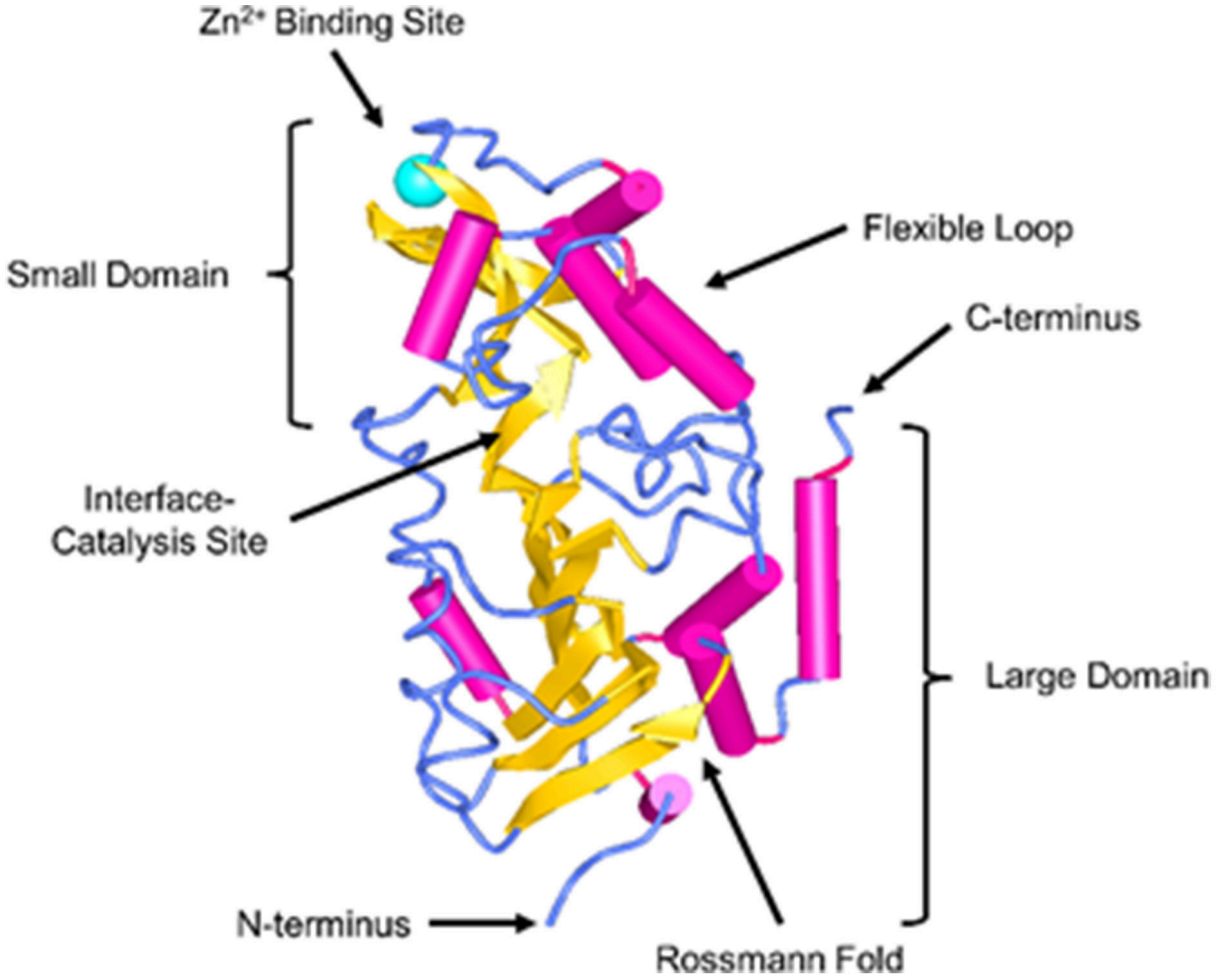
Structure of SIRT3. SIRT3 is depicted in the cartoon representation using NCBI Structure web-based 3D structure viewer and assembled from Protein Data Bank code 3GLU (12).

Sirtuins are a family of nicotinamide adenine dinucleotide (NAD^+^) dependent Class III histone deacetylases. They comprise of seven different proteins (Sirt1-7) and have been shown to regulate a broad extent of physiological and pathological processes, including energy production, stress resistance, reactive oxygen species (ROS), mitochondrial homeostasis, apoptosis, and aging (7–11). In recent years, there has been a growing interest in the cardioprotective effects of SIRT3. SIRT3 was initially reported to be primarily localized to the mitochondria. Human SIRT3 protein consists of 399 amino acids and has two functional domains: a large Rossmann fold and NAD^+^ binding site, and a small helical complex and zinc binding motif (Figure 1). The acetylated substrate is inserted into the cleft between these two domains (12). The full length of SIRT3 (44 kDa) is enzymatically inactive and is cleaved by mitochondrial matrix processing peptidase (MPP) during its translocation into the mitochondria, resulting in a shorter and active 28 kDa form. SIRT3 may correlate with longevity in humans implicated by the studies showing that the expression of SIRT3 was decreased in old sedentary adults compared to younger individuals and other populations studied (13, 14). SIRT3 is involved in the regulation of mitochondrial functions and cellular metabolism in energy-demanding cells, including fatty acid oxidation, tricarboxylic acid cycle (TCA) and the electron transport chain (9, 15–19). Despite the fact that SIRT3 regulates the core mitochondrial processes, its function may differ in fuel-producing and fuel-utilizing tissues depending on the specific metabolic pathway (20). Thus, SIRT3 may play diverse roles that involve tissue and cell specific functions. Studies have shown that SIRT3 deficiency in myoblast and cancer cells led to impaired mitochondrial respiration and increased ROS formation (21–23). Moreover, respiratory capacity and ATP synthesis were decreased in cardiac mitochondria of SIRT3 deficient mice (17).

SIRT3 has been shown to blockade cardiac hypertrophy and attenuate aging and oxidative stress-mediated cell death in cardiomyocytes via Foxo3a and Ku70 (24). In addition, SIRT3 deficiency impairs mitochondrial function and cardiac function by hyperacetylation of energy metabolic proteins and myocardial energy depletion (16, 17). While endothelial cells comprise the inner layer of the blood vessel wall and capillaries as well as a large proportion of cell population in the heart, interestingly, their metabolic status do not gain enough attention in relation to SIRT3. Although the role of SIRT3 on mitochondrial function has been extensively investigated, the metabolic profile associated with SIRT3 deficiency in EC has not been fully examined. In this issue of the research topic: **Cardiac Microvascular Endothelium Contribution to Cardiac Myocyte Growth, Structure, and Contractile Function**, we specifically discuss the emerging role of SIRT3 in EC glycolytic metabolism, microvascular rarefaction and HFpEF.

### SIRT3 and Endothelial Cell Metabolism

SIRT3 is primarily located in the mitochondria and functions to regulate energy metabolism and oxidative stress. However, the role of SIRT3 in EC glycolytic metabolism has not been intensively investigated. Emerging evidence suggests that ECs preferentially use glycolysis rather than oxidative phosphorylation to generate ATP in order to maintain their normal functions. We see this during angiogenesis, as EC sprouting, proliferation, and migration are all glycolysis dependent processes (25–28). ECs have lower oxygen consumption and mitochondrial content compared to other oxidative cell types, such as cardiomyocytes (29). As such, ECs have a higher glycolysis rate than cardiomyocytes, comparable to or higher than rates seen in cancer cells (25, 29). During angiogenesis, alteration of mitochondrial ATP production or mitochondrial respiration does not have an effect on vessel sprouting, whereas blockade of glycolysis by either limiting glucose availability or replacing with glucose analogue-2-deoxy-D-glucose (2-DG) leads to endothelial dysfunction and cell death (25, 30, 31). These studies indicate a critical role of glycolytic metabolism in regulating EC function. But how does SIRT3 affect EC metabolism? A recent study from our laboratory demonstrated that SIRT3 deficient ECs exhibited decreased basal glycolysis and glycolytic capacity (32). This data implicated an association between SIRT3 and EC glycolysis. However, the molecular mechanism by which SIRT3 regulates EC metabolism remains unclear. In quiescent states, ECs are mainly dependent on glycolysis for supporting vascular homeostasis and EC function. During angiogenesis, EC glycolytic metabolism is enhanced, in part, by upregulating the expression of 6-phosphofructo-2-kinase/fructose-2,6-bisphosphatase-3 (PFKFB3) (25, 29). A study revealed that pharmacologic inhibition of PFKFB3, a key glycolytic enzyme in EC, reduced glycolytic flux by about 40% in EC (25, 31). Consistent with this data, our studies showed that PFKFB3 was down-regulated via hyper-acetylation in SIRT3 deficient ECs, suggesting PFKFB3 may be the mechanistic link between SIRT3 and EC metabolism (32). Therefore, inhibition of PFKFB3 in EC cells led to decreased expression of angiogenic factors and increased expression of inflammatory cytokines, (32). Taken together, SIRT3 deficiency may be the cause of endothelial dysfunction by reducing the expression of PFKFB3, reprogramming EC metabolisms and promoting inflammation (24).

Although EC has a small bulk of mitochondria and mitochondria generate a small amount of ATP as compared to glycolysis, this does not suggest mitochondria are not important (33, 34). In fact, EC mitochondria have been shown to maintain EC homeostasis and function, and work as signaling organelles via the production of ROS (33, 34). SIRT3 is predominately localized in the mitochondria and regulate mitochondrial functions by deacetylation of several important metabolic enzymes. It has been shown that SIRT3 promotes ROS detoxification through deacetylation of antioxidant enzymes such as manganese superoxide dismutase (MnSOD) and catalase (CAT) (8, 18, 35). Intriguingly, our study demonstrated that ROS production was dramatically increased in SIRT3 deficient ECs, which was associated with elevated mitochondrial oxygen consumption rate (32). These data suggest that there is a metabolic reprogramming in SIRT3 deficient ECs with impaired glycolytic metabolic flexibility and prone to oxidative phosphorylation and mitochondrial ROS formation. Hypoxia-inducible factors (HIFs) play a central role in the hypoxia response pathway and mitochondria has been shown to regulate endothelial HIF-2α stabilization by ROS (33). Data from our laboratory revealed that SIRT3 deficiency downregulated hypoxia-induced expression of HIF-2α in ECs together with a significant reduction of angiopoietin-1 and VEGF expression, suggesting an impaired hypoxia signaling and angiogenesis possibly mediated by SIRT3/mitochondria/ROS pathway (36). Interestingly, treatment of ECs with HIFα inducer dimethyloxalylglycine (DMOG) rescued both the expression of PFKFB3 and glycolytic function (37). This treatment with DMOG also decreased the maximal mitochondrial oxygen consumption rate in SIRT3 deficient ECs (37), further supporting the functional role of HIF-2α/mitochondria in regulate EC metabolism. ECs prefer glycolysis as their primary energy source for the purposes of minimizing oxygen consumption, increasing oxygen delivery to the surrounding tissue, adapting to a low oxygen tension state, and generating ATP faster than with oxidative phosphorylation (29, 31, 38). However, our study showed that glycolysis was decreased, whereas oxygen consumption rate was increased in SIRT3 deficient ECs (32), implicating possible impairment of angiogenesis and response to stress.

### SIRT3 and Coronary Microvascular Dysfunction

Coronary microvascular dysfunction is evaluated by determination of coronary flow reserve (CFR) and impaired CFR is a powerful independent correlate of cardiac mortality and coronary microvascular rarefaction in patients with heart failure, especially HFpEF (39). Patients with diabetes have higher prevalence of impaired CFR (40). Preexisting coronary microvascular dysfunction in diabetes may contribute to the microvascular obstruction and no-reflow after percutaneous coronary intervention. Myocardial capillaries are the primary determinant of CFR (41). Microvascular rarefaction (reduced myocardial capillary density) and impairment of angiogenesis are considered as a major feature of HFpEF (26). Microvascular rarefaction reduced eNOS, lowered NO bioavailability and resulted in cardiomyocyte stiffness, cardiac hypertrophy and HFpEF (26, 42–44). Microvascular rarefaction also results in a decreased CFR rendering the heart vulnerable to hypoxia that led to increased ROS formation, cardiomyocyte death and heart failure with reduced ejection fraction (HFrEF) (26, 43, 45, 46). Emerging evidence reveals a critical role of sirtuin family in the cell metabolism and EC angiogenesis. Up to date, SIRT1 is the most well-investigated member of sirtuin family that has been shown to be involved in angiogenesis and be upregulated during neovascularization (47, 48). A study has shown that deficiency of SIRT1 in EC or specific knockout of endothelial cell SIRT1 in mice led to a significant reduction of EC sprouting and branching, followed by an impairment of ischemia-induced neovascularization (48). In contrast, treatment with resveratrol, a SIRT1 activator, has been shown to protect heart against myocardial ischemic injury by increasing capillary density via upregulation of VEGF and nitric oxide synthase (49). In addition, SIRT1 has been reported to promote angiogenesis via deacetylation and inactivation of p53 (50, 51).

Previous studies showed that overexpression of SIRT3 blocks cardiac hypertrophy whereas knockout of SIRT3 in aged mice promotes cardiac hypertrophy (52, 53). Emerging evidence suggest the regulatory roles of SIRT3 in the regulation of EC metabolism, angiogenesis and heart failure (24, 32, 36, 37). Bone marrow cell (BMC)-derived endothelial progenitor cells (EPCs) from SIRT3 deficient mice has been shown to have impaired angiogenic capacities and colony formation as well as increased ROS formation and apoptosis (54). Moreover, these abnormalities of SIRT3 deficient BMCs limited the BMCs-mediated cardiac repair in post-myocardial infarction (MI) mice (54). Similarly, apelin-mediated improvement of BMCs therapy on cardiac repair and cardiac function was abolished in the absence of SIRT3 in the post-MI mice (55). SIRT3 levels are also decreased in patients with type II diabetes (56–58). Diabetes exhibits microvascular rarefaction and is one of the strongest risk factors for development of HFpEF, and 85% of older HFpEF patients have a diabetic phenotype (1, 2). Our study demonstrated that the expression of SIRT3 was reduced in the heart of db/db diabetic mice together with microvascular rarefaction and impaired CFR. Angiogenic growth factor apelin gene therapy increased expression of SIRT3 and angiogenic growth factors, thus reduced myocardial microvascular rarefaction in the heart of db/db diabetic mice (59). In agreement with this finding, apelin gene therapy in STZ-induced diabetic mice showed an increased myocardial angiogenesis and alleviation of MI injury in diabetic STZ-wild-type (WT) mice, but not in STZ-SIRT3 KO mice (60). These studies implied a potential role of SIRT3 in the regulation of angiogenesis. Nonetheless, additional studies are needed to establish a comprehensive understanding of the molecular mechanisms by which endothelial SIRT3 deficiency regulates coronary microvascular rarefaction and dysfunction.

Recent studies have highlighted the role of SIRT3 on the angiogenic capacity of ECs, indicating that a deficiency of SIRT3 reduces endothelial proliferation, tube formation, and migration via impaired glycolytic function (32, 36). Consistent with this result, SIRT3 KO mice and SIRT3^EC^KO mice manifested reduced myocardial capillary density with impairment of CFR and diastolic function (32, 36). Other studies suggest a possible mechanism involving SIRT1 that selectively augments HIF-2α signaling during hypoxia (61). In addition, ECs are rarely sprout, migrate, or proliferate in quiescent, whereas they can sense and respond to hypoxia resulting in a shift to highly glycolytic metabolism and increase sprouting, migration and proliferation (15, 62). This phenotype change is primarily mediated by the activation of EC glycolytic metabolism such as activation of PFKFB3 signaling pathway (25, 28, 63, 64). Interestingly, impaired hypoxia-induced expression of HIF-2α was found in SIRT3 deficient ECs, and was associated with decreased hypoxia-induced expression of angiogpoietin-1 and VEGF (32, 37). This impaired hypoxia signaling further reduced tube formation by SIRT3 deficient ECs (32, 37). Nonetheless, the angiogenic capacity of SIRT3 deficient ECs was rescued after the treatment with HIF-inducer DMOG along with recovered endothelial glycolytic function (37). Consistent with these findings, SIRT3 KO mice also exhibit a lower level of HIF-2α and increased expression of prolyl hydroxylase PHD1, suggesting a potential mechanism of decreased myocardial capillary density in SIRT3 KO mice. Most importantly, treatment with DMOG restored impaired CFR and reversed pre-existing diastolic dysfunction in SIRT3 KO mice and SIRT3^EC^KO mice (37). These results demonstrated that improvement of EC glycolytic metabolism can reverse the pre-existing diastolic dysfunction in SIRT3 deficient mice and SIRT3^EC^KO mice. This study provides a potential therapeutic strategy of targeting EC glycolytic metabolism for patients with HFpEF associated with coronary microvascular rarefaction, especially in the aging population with reduced SIRT3.

### SIRT3 and Heart Failure

Recent study has shown that knockout of SIRT3 enhances weight gain and reduces rapid metabolic adaptation in LDL receptor KO mice, implicating a critical role of SIRT3 in delaying the development of atherosclerosis (65). SIRT3 levels were decreased within advanced age, cardiovascular and metabolic diseases. HFpEF is strongly associated with advanced age, hypertension and metabolic syndrome (7, 9, 26, 66). The results from our studies showed that SIRT3 deficient mice have normal cardiac function at a young age but subsequently develop a HFrEF phenotype at 12 months of age compared to age-matched WT mice (32, 37). These findings indicated that SIRT3 are key regulators for maintaining normal heart function during aging and in the development of aging-associated heart failure. So far, two heart failure phenotypes are defined, namely, heart failure with reduced ejection fraction (HFrEF) and heart failure with preserved ejection fraction (HFpEF). In clinic, almost half of the population of patients with HF are HFpEF patients (1, 2). Although the standard-of-care of medications for HFrEF with angiotensin-converting-enzyme inhibitors (ACEIs), angiotensin receptor blockers (ARB), beta-blockers, and statins treatments have been well-established, no comparable effective treatment has been identified in randomized clinical trials in HFpEF (6, 45, 67–71). The reasons for the failure of HFpEF is unknown at present. However, HFpEFs that are strongly associated with obesity, hypertension, diabetes mellitus and chronic kidney disease, may result in systematic inflammation including coronary EC that causes coronary microvascular dysfunction and decreased bioavailability of NO, impairment of CFR which leads to impaired diastolic function (46). In line with these, clinical studies also revealed that HFpEF patients exhibit abnormalities in coronary microcirculation related to endothelial dysfunction and coronary microvascular rarefaction (26). Coronary blood flow is dependent on EC derived NO and endothelial dysfunction and decreased microvascular density may reduce CFR (26). Excessive production of ROS can further decrease nitric oxide bioavailability to the cardiomyocytes (72). Consistent with these findings, our study demonstrated that ROS production was dramatically increased in SIRT3 deficient ECs and SIRT3 KO mice exhibited decreased capillary density and developed coronary microvascular dysfunction as evidenced by a reduction of CFR, which resulted in worse cardiac function and impaired post-MI cardiac recovery compared to WT mice (36). A possible mechanism is that SIRT3 deficiency-induced metabolic dysfunction and an increase in ROS in ECs result in endothelial dysfunction which limits coronary blood flow in response to increased metabolic demand or ischemia (32, 37). SIRT3 KO mice also developed diastolic dysfunction as evidenced by prolonged isovolumic relaxation time (IVRT) and increased myocardial performance index (MPI) (32, 37). These findings were also associated with impairment of HIF-2α signaling (32, 37). Intriguingly, treatment of SIRT3 KO mice with DMOG rescued the CFR, diastolic function and systolic function(37). Indeed, HIF-2α deficiency is associated with disruption of ROS homeostasis and cardiac hypertrophy that is a risk factor of diastolic dysfunction (73).

**Figure 2:**
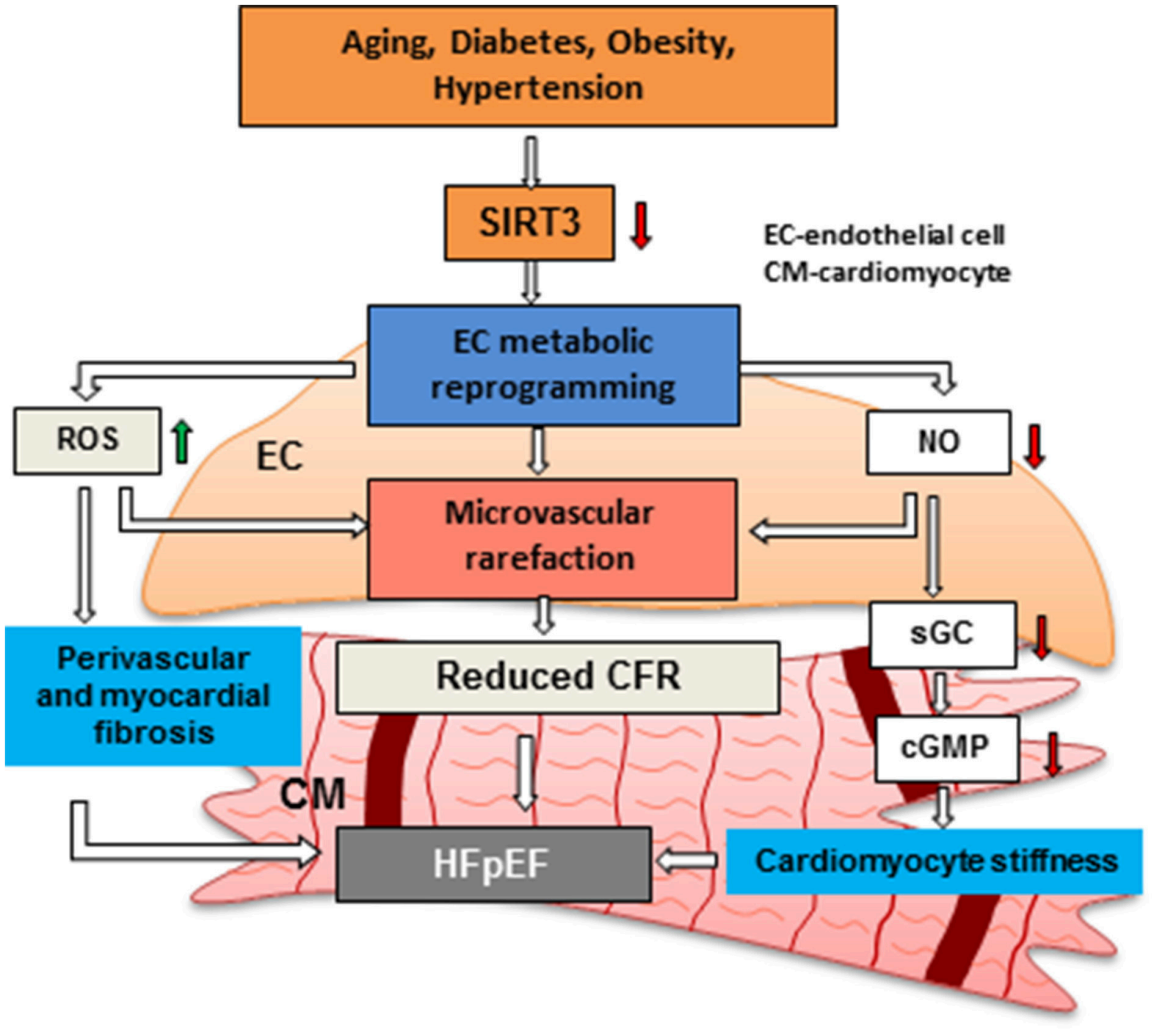
Hypothesis regarding the role of endothelial SIRTUIN 3 and microvascular rarefaction in the pathophysiology of HFpEF. Risk factors, such as aging, diabetes, obesity and hypertension has been shown to reduce the expression of SIRT3 in EC. Loss of SIRT3 shifts ECs from oxygen-independent metabolism to highly oxygen-consuming metabolism. This metabolic reprogramming in EC increases oxygen demand and induces production of ROS, thus results in an increase in cardiomyocyte fibrosis. Disruption of endothelial glycolytic metabolism also leads to impairment of angiogenesis and microvascular rarefaction. In addition, impairment of NO production promotes cardiomyocyte stiffness by reducing sGC/cGMP. All these critical steps may lead to microvascular rarefaction and diastolic dysfunction and HFpEF. SIRT3: Sirtuin 3, CFR: coronary flow reserve, EC: endothelial cell, CM: cardiomyocyte, HFpEF: heart failure with preserved ejection fraction, NO: nitric oxide.

Most of the aforementioned findings were demonstrated in global SIRT3 deficient mice in which all cell types, such as cardiomyocytes, EC, pericytes and fibroblasts, can contribute to the development of coronary microvascular, diastolic and systolic dysfunction. Therefore, endothelial-specific SIRT3 knockout (ECKO) mice were developed and their cardiac function was assessed. Similarly, SIRT3 ECKO mice developed diastolic dysfunction along with decreased CFR, but without reduced systolic function probably due to the younger age of the mice (6–8 months old other than 12 months old) (32, 37). These findings suggest that endothelial SIRT3 and coronary microvascular dysfunction play an important role in the development of HFpEF. We hypothesize that SIRT3 deficiency increases ROS and increases perivascular and myocardial fibrosis, thus resulting in endothelial and myocardial dysfunction. Reduction of NO production in EC limits NO delivered to the cardiomyocytes which leads to an impairment of sGC/cGMP pathway and increased cardiomyocyte stiffness. SIRT3 deficiency in EC also alters glycolytic metabolism, resulting in metabolic reprogramming from an oxygen-independent to a highly oxygen-consuming metabolism. This further increases oxygen demand and decreases oxygen delivered to cardiomyocytes in coordination with decreased coronary capillary density and cardiomyocyte hypoxia. All these alterations mediated by loss of SIRT3 in EC lead to microvascular rarefaction, impaired CFR, myocardial stiffness and HFpEF (Figure 2).

## Conclusion

SIRT3 has great potential in the modulation of a variety of cellular processes, through the link between protein acetylation and various physiological functions and diseases. Although SIRT3-mediated cardiovascular diseases have been intensively investigated and our understanding of the biology of SIRT3 has expanded remarkably over the past decade (24), the role of SIRT3 in endothelial metabolism and coronary microvascular dysfunction has not been well-studied. Recent studies revealed that SIRT3 is critical for EC glycolysis and angiogenesis, and Sirt3 deficiency predisposes to coronary endothelial dysfunction and increases the risk for developing HF. Although the underlying molecular mechanism of SIRT3 on endothelial metabolic reprogramming is still incomplete, it is clear that endothelial SIRT3 deficiency contributes to the accelerated development of age-related heart failure with preserved ejection fraction. So far, there is no clinical trial to explore the therapeutic role of SIRT3 due to lack of SIRT3 specific activators. Therefore, it is urgent to develop new agents which specifically target mitochondrial SIRT3 in the future. A few small molecules, such as resveratrol and honokiol have also been shown to activate SIRT3 or its downstream targets and thus provide potential strategy for cardiac therapeutics. From this perspective, the studies discussed here just scratch the surface of the whole picture of endothelial SIRT3; further investigations are warranted to address the intracellular molecular mechanisms mediated by endothelial SIRT3 and discover new SIRT3 specific agonists and potential therapeutic approaches for targeting EC glycolytic metabolism in the treatment of HFpEF.

## Author Contributions

All authors listed have made a substantial, direct and intellectual contribution to the work, and approved it for publication.

### Conflict of Interest Statement

The authors declare that the research was conducted in the absence of any commercial or financial relationships that could be construed as a potential conflict of interest.
